# From Day to Day

**Published:** 1888-03-10

**Authors:** 


### The Hospital

A Weekly Institutional Journal of

Science, Medicine, and Philanthropy.

*For Hospitals, Asylums, Medical Practitioners, Students, Nurses, Families, Parishes, Congregations, and the Charitable Public.*

Vol. III. No. 76.] [March 10, 1888.

### From Day to Day.

At the present crisis almost every man and woman in Europe and America who reads a daily newspaper turns first to the column which gives him the latest information about the German Crown Prince. Each one watches the case from day to day with curiosity, with anxiety, and with perplexity. That the Prince is an illustrious person, and that tremendous possibilities depend upon his life, account for much of the eager interest displayed by men of all ranks; but the difficulties and uncertainties of the case, and the strange and conflicting accounts which reach the public almost every succeeding hour, have heightened anxiety to a tension which is painful. The general public cannot understand why the medical men are not more sure of their diagnosis, and why they cannot prognosticate with more definiteness the probable result. It is practically certain that in their inmost hearts the physicians and surgeons who are in charge of the case are all agreed: they are agreed to this extent at least, that there is an affection of the throat which is accompanied by inflammation and destruction of tissue, and that the destructive process is continuing in spite of all their efforts to check it. The point of uncertainty, not necessarily of divergence, is whether or not there has been a growth of new tissue of a cancerous nature. At the time of writing there has been no tissue removed by operation, or brought up by expectoration, of which reliable microscopists have been able to say with absolute certainty, "This is cancerous tissue—these are cancer cells." For want of this positive and convincing fact, it has been impossible for the responsible attendants to say with definite precision that the disease was or was not cancer.

Many people in this country and many more in Germany are ready to accuse Sir Morell Mackenzie either of want of knowledge or of want of candour. There is no justification for either accusation, as medical men very well know. At the outset the physician may well have hoped that the affection was not cancerous. There are other growths as well as those of a cancerous nature which affect the larynx and other parts of the throat. In the early stages of certain new growths it is frequently quite impossible to say with scientific accuracy what they may ultimately prove to be. The difficulty is increased in cases where the growths are small, and where it is impossible to obtain a portion for microscopic examination which has reached such a stage of development as to reveal its true structure with certainty. A medical man may, and often does, fear that a given growth will finally prove cancerous; but then he knows that it may not prove so; and knowing this, what kind of man would he be if he announced a positive diagnosis of cancer on the strength of a mere probability? The word "cancer" is such a terrible and dreadful harbinger of death that no man is justified even in naming it to his patient until he is either absolutely convinced that cancer is present, or satisfied that an operation is desirable, and finds it necessary to hint at cancer in order to obtain the patient's consent to active treatment. So long as there is a doubt, so long does the judgment of the physician as well as the instinct of the man plead for the more hopeful view. Whilst doubt continues, hope continues; and hope is more effectual in maintaining the general health and happiness of the patient than all the other remedies of the physician's armamentarium.

Should the disease in the present instance finally prove to be cancerous and fatal, many persons will think the German physicians justified in their doubtfully courteous treatment of the English specialist. There will be no justice or even sense in such a view. Whilst men of world-wide histological fame like Virchow have declined to give a positive verdict of cancer, a throat specialist, who has never claimed to be an authoritative microscopist, has had no choice but to concur in the uncertainty. He would have been unworthy of the name of scientist had he done otherwise. We repeat, that whatever his fears and anticipations might be, he was shut up to the line which at the outset he properly took, and to which he has steadily adhered. There is no intention on our part to make special pleading for Sir Morell Mackenzie because he is an Englishman or for any other reason. Justice and science alike demand that he shall be held clear of the odium of want of professional knowledge on the one hand, or of failure in honourable conduct on the other.

The case of the Crown Prince is one which teaches important lessons both to the public and to medical men. Physicians may learn from it the danger and folly of jumping rapidly to conclusions. It is a not uncommon habit with some practitioners, especially those in family practice, to announce a diagnosis almost at the first interview. Such a diagnosis once positively made, the rash physician finds a difficulty in withdrawing from it. There are few things any of us admit to our patients so unwillingly as a mistake in the nature of a disease. And yet if we have been mistaken, what criminal folly is it, and what direful consequences may result, if the mistake be persisted in! Better a thousand times admit the error in judgment, and even lose the family, than lose our own honour, and perhaps the patient's life. But all such troubles may be avoided by care, circumspection, and candour at the outset. Every family physician ought to have his clients so trained and instructed that they will not press him, in the early stage of attendance for a diagnosis, but will wait until he volunteers one of his own accord. This may be difficult among the poor and ignorant, though even by them an honest and capable man is soon trusted; but amongst the intelligent classes the tried and proved medical attendant often finds his patients quite ready to sympathise with his critical uncertainty, and to wait any reasonable length of time for positive information.

The lesson taught by the case of the Crown Prince to those who employ medical men is still more emphatic than that impressed upon physicians. There are a certain class of patients who are the bane and terror of the doc'or's life. No sooner is he inside the house than they expect to know at once what is the matter, why the disease has come, how long they will be ill, whether they will get well or die, and every possible contingency which may arise during the whole conduct of the case. If the doctor is a nervous and foolish man he often commits himself to opinions which the after-history entirely fails to justify, and so is discredited for ever. If he does not commit himself, but asks for time, he is held to be ignorant and unequal to the emergency. If the plain truth must be spoken of such people, they are downright fools. There are hundreds of different diseases which affect mankind. Many of them begin with symptoms so similar as to be almost identical. These similar symptoms will often continue for days before the distinctive characteristics of the disease show themselves; sometimes they may even continue for weeks. How can the doctor say in such a case what the ultimate result may be? If he does hazard a guess, he may be wrong almost as often as he is right. Besides, he does no good by hazarding a guess—it does not help his treatment. He merely gratifies a momentary curiosity, which, though not unnatural, is not intelligent, and ought to be repressed.

Here is the lesson the world should learn: The Crown Prince of one of the greatest empires under the sun is ill of a dangerous malady. The wisest and the most practically efficient modern science has not as yet availed to arrest the disease or even to say with certainty what is its nature. All the impatience of all civilised peoples does not alter the facts in the smallest degree. Medical opinions are being asked daily all the world over; and still the disease holds on its way, and nothing effectual has been done to stay its advance. If that be so in the case of the heir to the mighty monarchy of Germany, is it wonderful that among less distinguished persons there are diseases which less eminent scientists also fail either to specify distinctly or to cure? Men have long made up their minds that religious teachers, legal luminaries, politicians, and philosophers are limited, fallible, and often powerless either to pronounce with certainty or to act with success. They have now to see that physicians and scientists, even of the highest degree, are merely men—limited, conditioned, and not seldom incapable of saying one authoritative word or saving one single life. Honest physicians, like other honest men, give all they can to the world. When they have given that they stand aside and wait. The world will be wise to expect no more from them than that which is possible to men.

